# Long noncoding RNA lnc-LOC645166 promotes adriamycin resistance via NF-κB/GATA3 axis in breast cancer

**DOI:** 10.18632/aging.103012

**Published:** 2020-05-27

**Authors:** Ruinian Zheng, Jun Jia, Ling Guan, Huiling Yuan, Kejun Liu, Chun Liu, Weibiao Ye, Yuting Liao, Shunhuan Lin, Ou Huang

**Affiliations:** 1 Department of Oncology, Affiliated Dongguan People's Hospital, Southern Medical University, Dongguan 523000, Guangdong Province, P.R. China; 2 Clinical Research Center, Affiliated Dongguan People's Hospital, Southern Medical University, Dongguan 523000, Guangdong Province, P.R. China; 3 Department of Galactophore, Affiliated Dongguan People's Hospital, Southern Medical University, Dongguan 523000, Guangdong Province, P.R. China; 4 Department of Pathology, Affiliated Dongguan People's Hospital, Southern Medical University, Dongguan 523000, Guangdong Province, P.R. China; 5 Comprehensive Breast Health Center, Ruijin Hospital, Shanghai Jiaotong University School of Medicine, Shanghai 200025, P.R. China

**Keywords:** chemoresistance, lncRNA-LOC645166, breast cancer, GATA3, NF-κB

## Abstract

Chemoresistance remains a significant obstacle for effective adriamycin (ADR) treatment in breast cancer. Recent efforts have revealed that long noncoding RNAs (lncRNAs) play a crucial role in cancer biology, including chemoresistance. We identified the lncRNA LOC645166 was upregulated in adriamycin resistant-breast cancer cells by Microarray analysis, which was further confirmed in the tissues of nonresponsive patients by reverse transcription-quantitative polymerase chain reaction (RT–qPCR), western blotting, and immunohistochemical assays. Downregulation of lncRNA LOC645166 increased cell sensitivity to adriamycin both *in vitro* and *in vivo*. In contrast, upregulation of lncRNA LOC645166 strengthened the tolerance of breast cancer cells to adriamycin. Chromatin immunoprecipitation (ChIP) and RNA binding protein immunoprecipitation (RIP) demonstrated that lncRNA LOC645166 could increase the expression of GATA binding protein 3 (GATA3) via binding with nuclear factor kappa-light-chain-enhancer of activated B cells (NF-κB), leading to the activation of STAT3 and promoting chemoresistance in breast cancer. Together, the present study suggested that lncRNA LOC645166 mediated adriamycin chemoresistance in breast cancer by regulating GATA3 via NF-κB.

## INTRODUCTION

Breast cancer is one of the most common cancers diagnosed in women. Breast cancer can occur in both men and women, but is far more common in women, making breast cancer the number one cause of death in women diagnosed with a cancer-related disease worldwide [1]. Although profound advances have been achieved, the survival rate of patients with breast cancer remains unsatisfactory. Chemotherapy is the standard treatment option for advanced breast cancer [2]; however, drug resistance has become the primary obstacle for breast carcinoma therapy [3]. Understanding the molecular mechanisms involving chemoresistance and developing effective strategies are urgently needed. Adriamycin (ADR) is from the anthracycline and antitumor antibiotic family of medications, which interacts with DNA by intercalating and inhibiting macromolecular biosynthesis. Through a series of steps, ADR stops the process of DNA replication by intercalating between two base pairs of the DNA double helix [4]. ADR efficiently reduces the recurrence and mortality in breast cancer patients. However, several patients have failed to respond to treatments with ADR, owing to an intrinsic or acquired resistance [5]. In addition, reliable biomarkers that may predict drug responses are not currently available.

Long noncoding RNAs (lncRNAs) are a group of transcripts with a minimum length of 200 nucleotides (nt) and limited protein-coding ability [6, 7]. Accumulating evidence has indicated that the aberrant expression of lncRNAs has been demonstrated in multiple malignancies, and is closely associated with cellular functions as well as participates in pathological tumour processes such as cancer cell growth, metastasis, and chemoresistance [8–11]. Up to now, several lncRNAs have indicated lncRNAs have roles in drug resistance. The lncRNA forkhead box protein C2-antisense RNA 1 (*FOXC2-AS1*) promotes doxorubicin-resistance of osteosarcoma by modulating the expression of *FOXC2* [12]. The lncRNA bladder and prostate cancer suppressor (*LBCS*) inhibits chemoresistance of bladder cancer stem cells by epigenetically silencing SRY-box transcription factor 2 (*SOX*). The lncRNA hepatocellular carcinoma up-regulated long non-coding RNA (*HULC*) increases the chemoresistance of hepatocellular carcinoma (HCC) cells by triggering autophagy [13]. The maternally expressed 3 (*MEG3*) lncRNA regulates cisplatin-resistance in lung adenocarcinoma cells by regulating p53 and B-cell CLL/lymphoma 2 (*BCL2*) expression [14]. However, the role of lncRNAs in the resistance of breast cancer to adriamycin is not yet to be fully understood. Thus, this study aims to investigate the contributions of lncRNA to adriamycin tolerance in breast cancer, and to evaluate the clinical significance of adriamycin-resistant patients with breast cancer.

## RESULTS

### lncRNA LOC645166 is preferentially upregulated in breast cancer with adriamycin-resistance

**Figure 1:**
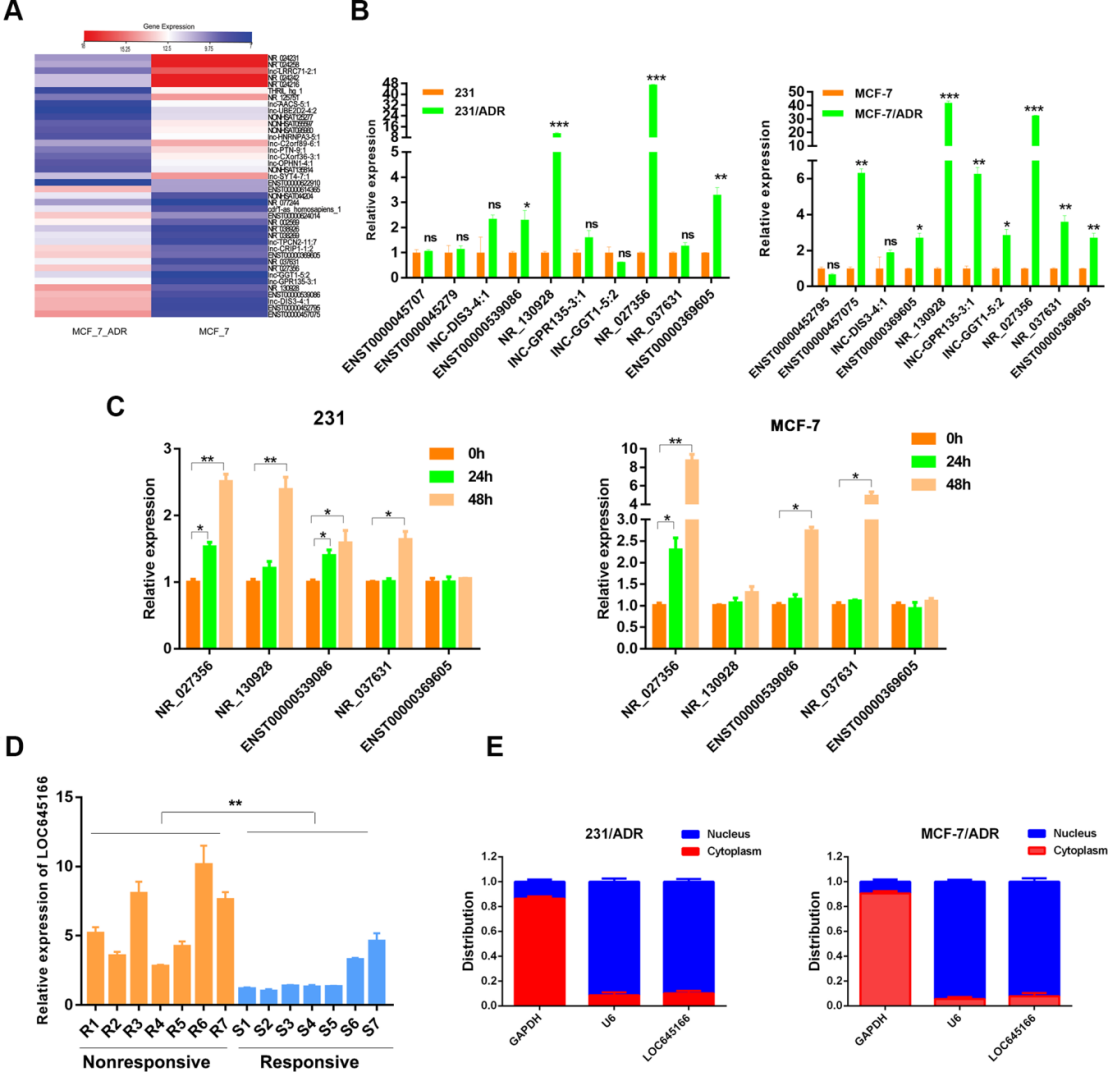
**lnc-LOC645166 is upregulated in breast cancer with adriamycin resistance.** (**A**) Heat map of differentially expressed lncRNAs (the top 20 in upregulated lncRNAs and the top 20 in downregulated lncRNAs) between MCF-7 and MCF-7/ADR cells. (**B**) RT-qPCR analysis of top 10 upregulated lncRNAs between ADR-resistant breast cancer cell lines and their parent cell lines (n =3). (**C**) RT-qPCR analysis of five different expressed lncRNAs after treated with ADR for 24h and 48h in MCF-7 and 231 cell lines (n =3). (**D**) RT-qPCR analysis of lnc-LOC645166 in an independent set of breast tumors samples with good or poor responses to ADR therapy (n =3), R means resistant to ADR therapy, S means sensitive to ADR therapy. (**E**) Subcellular fractionation of MCF-7/ADR and 231/ADR cells to determine the cellular location of lnc-LOC645166. GAPDH acted as cytoplasmic marker and U6 acted as nuclear marker (n=3). Data are shown as means ± SD. *p < 0.05, **p < 0.01, ***p < 0.001.

An lncRNA microarray analysis in a Michigan Cancer Foundation-7 (MCF-7) cell and an MCF-7 adriamycin-resistant cell (MCF-7/ADR) was applied to determine potential lncRNAs responsible for adriamycin unresponsiveness. A heatmap describing the changes in lncRNAs is shown in Figure 1A. Among the deregulated lncRNA transcripts (fold change ≥ 2), the top 10 lncRNAs showing upregulated expression were selected and subjected to validation via reverse transcription-quantitative polymerase chain reaction (RT–qPCR) using two paired adriamycin-resistant breast cancer cells and their parental cells (Figure 1B and Supplementary Figure 1). In total, five lncRNAs with increased expression in adriamycin-resistant cells were used for further screening. After treatment with adriamycin for 24 and 48 h, lncRNA LOC645166 (NR_027356) showed the most substantial increase in both MCF-7 and MDA-MB-231 cells (Figure 1C). Meanwhile, the expression of lncRNA LOC645166 was preferentially upregulated in adriamycin-nonresponsive patients compared to responsive patients (Figure 1D). The subcellular distribution assay revealed that lncRNA LOC645166 is predominately located in the nucleus (Figure 1E). We then analysed the potential role of lncRNA LOC645166 in adriamycin tolerance.

### lncRNA LOC645166 is required for adriamycin tolerance in breast cancer cells

**Figure 2:**
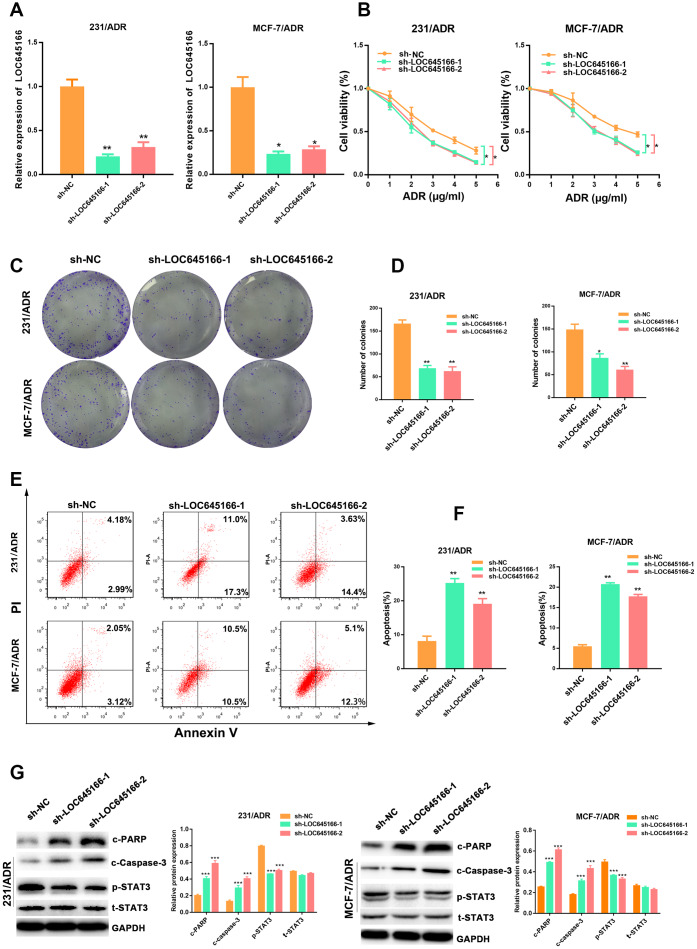
** lnc-LOC645166 enhanced the chemoresistance of breast cancer cells by affecting cell proliferation and apoptosis.** (**A**) RT–qPCR analysis of lnc-LOC645166 in 231/ADR and MCF-7/ADR cells transfected with sh-LOC645166-1, sh-LOC645166-2 or sh-NC for 48 h (n=3). (**B**) The effect of LOC645166 knockdown on the cell sensitivity to ADR of 231/ADR and MCF-7/ADR cells. (**C**, **D**) The effect of LOC645166 knockdown on the cell colony formation ability of 231/ADR and MCF-7/ADR cells under ADR treatment (231/ADR, 0.5μg/ml; MCF/ADR, 1μg/ml). (**E**, **F**) Flow cytometry analysis of Annexin V-stained 231/ADR and MCF-7/ADR cells transfected with sh-LOC645166-1, sh-LOC645166-2 or sh-NC upon adriamycin treatment (231/ADR, 0.5μg/ml; MCF/ADR, 1μg/ml) for 48 h (n=3). (**G**) Western blot analysis of the c-PARP,c-Caspase-3, p-STAT3 and t-STAT3 expression in 231/ADR and MCF-7/ADR cells transfected with sh-LOC645166-1, sh-LOC645166-2 or sh-NC upon adriamycin treatment (231/ADR, 0.5μg/ml; MCF/ADR, 1μg/ml) for 48 h (n=3). Data are shown as means ± SD. *p < 0.05, **p < 0.01, ***p < 0.001.

To investigate the effect of lncRNA LOC645166 on the chemoresistance of breast cancer, we transfected an MDA-MB-231 adriamycin-resistant cell lines (231/ADR) and MCF-7 adriamycin-resistant cell lines (MCF-7/ADR) with lncRNA LOC645166-specific short hairpin RNAs (shRNAs) or control shRNA (Figure 2A). Compared with the control group, silencing lncRNA LOC645166 re-sensitised breast cancer cells to adriamycin treatment (Figure 2B and Supplementary Figure 2A). In addition, the colony formation ability was inhibited in lncRNA LOC645166-knockdown breast cancer cells following adriamycin treatment (Figure 2C, 2D). Moreover, flow cytometry showed that adriamycin exposure resulted in an increased apoptosis rate of lncRNA LOC645166-knockdown cells (Figure 2E, 2F). In addition, lncRNA LOC645166 knockdown lead to the increased expression of cleavage of poly (ADP-ribose) polymerase (PARP), cleaved-caspase 3 (Figure 2G) and decreased expression of the phosphorylation of STAT3, as well as the promotion of caspase3 activity (Supplementary Figure 2B. Together, these data indicated that lncRNA LOC645166 is required for the adriamycin resistance observed in breast cancer cells.

**Figure 3:**
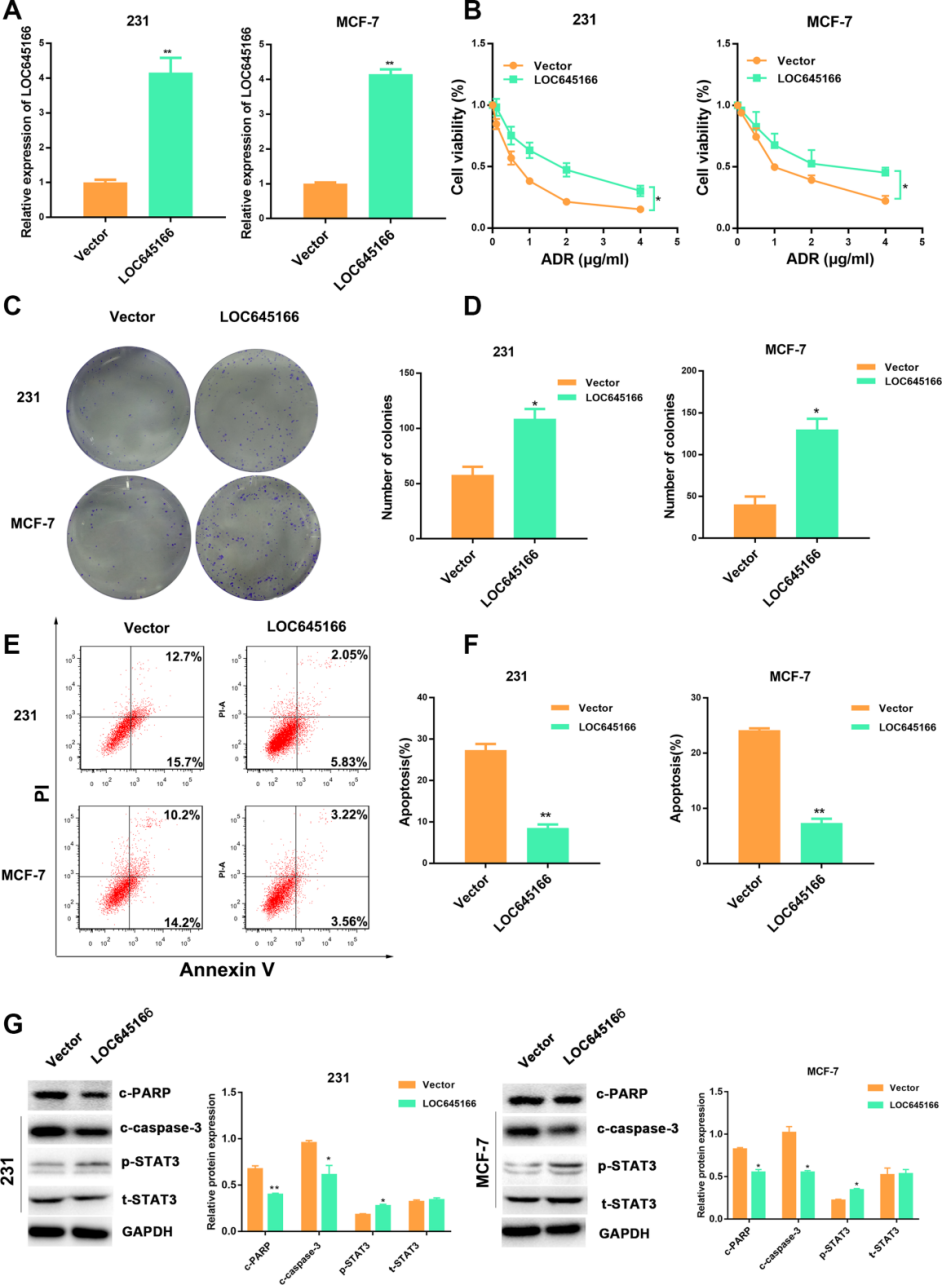
**Overexpression of lncRNA-LOC645166 confers adriamycin tolerance to breast cancer cells.** (**A**) RT–qPCR analysis of LOC645166 in 231 and MCF-7 cells transfected with Vector and lnc-LOC645166 for 48 h (n=3). (**B**) The effect of lnc-LOC645166 overexpression on the cell sensitivity to ADR of 231 and MCF-7 cells. (**C**, **D**) The effect of LOC645166 overexpression on the cell colony formation ability of 231 and MCF-7 cells under ADR treatment (231, 0.1μg/ml; MCF-7, 0.2μg/ml). (**E**, **F**) Flow cytometry analysis of Annexin V-stained 231 and MCF-7 cells transfected with Vector and lnc-LOC645166 upon adriamycin treatment (231, 0.1μg/ml; MCF-7, 0.2μg/ml) for 48 h (n=3).(**G**) Western blot analysis of the c-PARP,c-Caspase-3,p-STAT3 and STAT3 expression in 231and MCF-7 cells transfected with vector and lnc-LOC645166 upon adriamycin treatment (231, 0.1μg/ml; MCF-7, 0.2μg/ml) for 48 h (n=3). Data are shown as means ± SD. *p < 0.05, **p < 0.01, ***p < 0.001.

To determine whether the overexpression of lncRNA LOC645166 could promote adriamycin tolerance to breast cancer cells, we overexpressed lncRNA LOC645166 via the transfection of pcDNA-lncRNA LOC645166 (Figure 3A). CCK-8 assays and MDR1 expression showed lnc-LOC645166 led to an increased tolerance to adriamycin compared with the control cells (Figure 3B and Supplementary Figure 2C). In addition, lncRNA LOC645166 overexpression promoted the colony formation ability under adriamycin treatment (Figure 3C, 3D). Moreover, increased lncRNA LOC645166 attenuated adriamycin-induced cell apoptosis, which was confirmed by flow cytometry assays, apoptosis-related protein detection (Figure 3E–3G) and the caspase3 activity (Supplementary Figure 2D). Consistently, increased p-STAT3 expression was detected in lncRNA LOC645166 overexpression cells under adriamycin exposure. Collectively, these results indicated that the elevated expression of lncRNA LOC645166 raises the adriamycin tolerance of breast cancer cells.

### Knockdown of lnc-LOC645166 suppressed the growth and metastasis of ADR-resistant breast cancer cells *in vivo*

**Figure 4:**
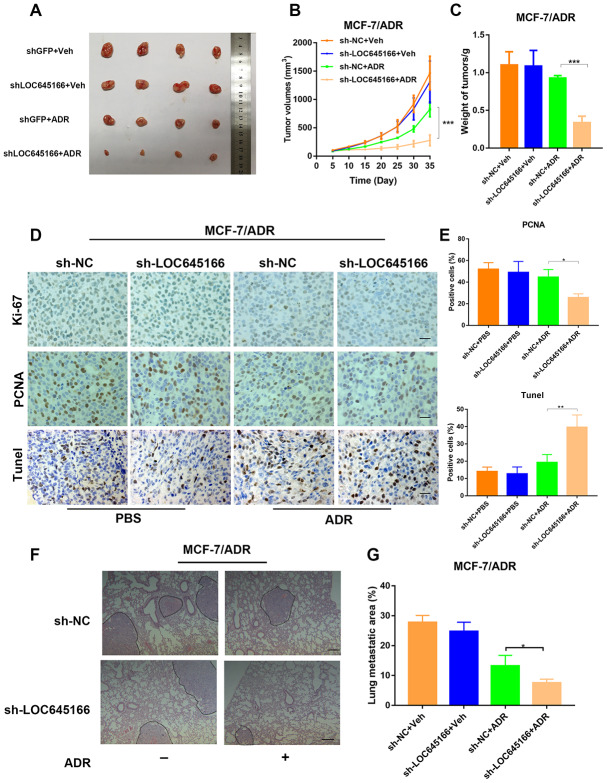
**Inhibition of lnc-LOC645166 suppresses the growth and metastasis of ADR-resistant breast cancer cells *in vivo*.** (**A**) Nude mice were subcutaneously xenografted with transfected lnc-LOC645166 knockdown and control adriamycin resistance breast cancer cells (MCF-7/ADR) and intraperitoneally injected with adriamycin every other day. Representative images of tumors and tumor volumes are shown. (**B**)Tumors derived from cells transfected with sh-LOC645166 or sh-NC were measured under a condition with PBS or adriamycin. Tumor volume was measured. (**C**) Weight of tumors derived from each group were calculated. (**D** and **E**) The tumor sections were subjected to H&E and immunostaining of Ki67 and TUNEL staining. (**F**) Representative HE images of metastatic lung in each group. Scale bar, 100 mm. (**G**) lung metastasis areas in each group. The data represented the mean±SD of three independent experiments. *P<0.05, **P<0.01, ***P<0.001.

To verify the effect of lncRNA LOC645166 on adriamycin resistance **in vivo**, we soundly transfected MCF-7/ADR cells with shRNA LOC645166 and shRNA NC. In total, 2×10^6^ cells were subcutaneously transplanted into nude mice, followed by adriamycin treatment. Cell growth was recorded by measuring the tumour volume every 5 days. A significant decrease in tumour growth was observed between lncRNA LOC645166 knockdown and control cells upon adriamycin treatment, while there was no observable difference between the lncRNA LOC645166 silenced group and the control group without adriamycin administration (Figure 4A–4C), indicating lncRNA LOC645166 may function as an adriamycin regulator in drug treatment.

Moreover, MCF-7/ADR cells steadily transfected with shRNA LOC645166 showed a reduced Ki-67 protein and proliferating cell nuclear antigen (PCNA) protein positivity staining under adriamycin treatment (Figure 4D, 4E). In addition, results from the transferase dUTP nick-end labelling (TUNEL) staining, revealed xenografts derived from MCF-7/ADR cells stably transfected with sh-LOC645166 presented with a significantly increased apoptosis rate compared to control cells during exposure to adriamycin (Figure 4D, 4E). Moreover, we established an animal model of lung metastasis via injecting transfected MCF-7/ADR into the tail vein of nude mice. When administered with adriamycin, lncRNA LOC645166 knockdown reduced the metastatic area compared to the control group, as verified by H&E stained lung sections (Figure 4F, 4G). Collectively, lncRNA LOC645166 knockdown remarkably hindered tumour growth and metastasis when treated with ADR.

### LncRNA LOC645166 promotes adriamycin resistance via GATA3

**Figure 5:**
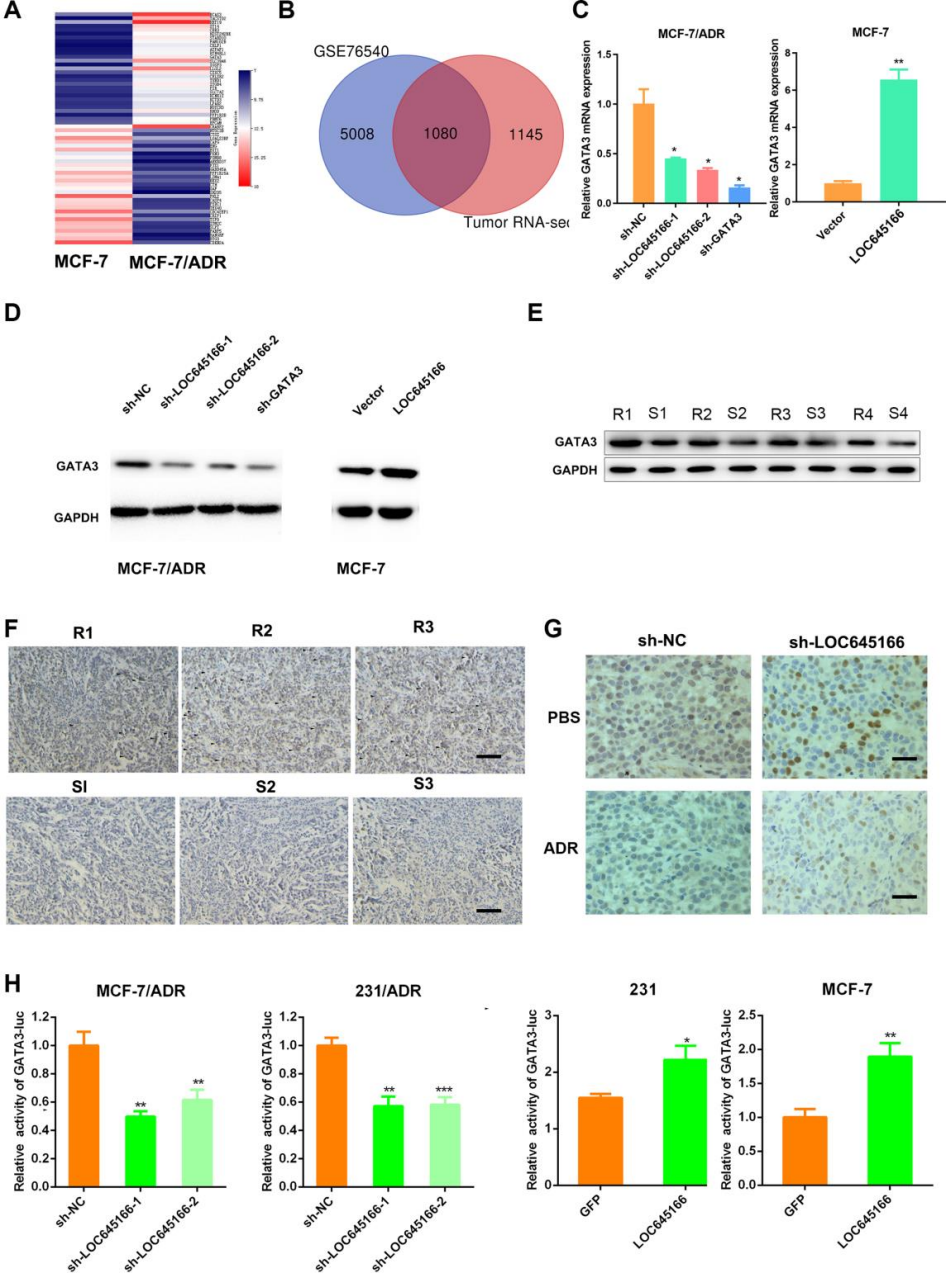
** lnc-LOC645166 promotes adriamycin resistance by activating GATA3.** (**A**) A heatmap of top ADR-resistant genes. (**B**) Common ADR-resistant genes by microarray and GEO database were selected as potential target genes. (**C**) RT–qPCR analysis of GATA3 when lnc-LOC645166 was silenced or overexpressed. (**D**) Western blots analysis of the GATA3 when lnc-LOC645166 was silenced or overexpressed. (**E**) Western blots analysis of the GATA3 protein in tumors derived from adriamycin resistance and sensitive patients. R means the resistance, S means sensitive. (**F**) Representative immunostaining of GATA3 in an independent set of tumor samples with good or poor responses to adriamycin therapy. R means resistant to ADR therapy, S means sensitive to ADR therapy. Scale bar=100 μm (**G**) Representative immunostaining of GATA3 in the transplanted tumors. Scale bar=50μm. (**H**) Luciferase activity assay of GATA3 luciferase reporter plasmids in breast cancer cells when lnc-LOC645166 was knockdown or overexpressed. The data represented the mean±SD of three independent experiments. *P<0.05, **P<0.01, ***P<0.001.

To investigate the downstream targets of lncRNA LOC645166, we referred to the microarray analysis of differently expressed genes between MCF-7/ADR and its parental cell, MCF-7. Because of the insufficient number of samples of our group, a GEO set of global differences of gene expressions between MCF-7 and MCF-7/ADR cells was also included. A heatmap revealed the top 15 genes with high and low expression levels, within the collective genes of the above two gene sets (Figure 5A, 5B). The top 15 genes were subjected to validation via RT–qPCR when lncRNA LOC645166 was silenced or overexpressed. As shown in Supplementary Figure 3A, GATA3 was upregulated in lncRNA LOC645166-overexpressed cells and downregulated in the lncRNA LOC645166 silenced group. RT–qPCR and Western blot assays further verified the regulation of lncRNA LOC645166 on GATA3(Figure 5C, 5D).

Furthermore, adriamycin-nonresponsive breast tissues had high GATA3 levels (Figure 5E, 5F) as verified by western blot assays and immunostaining. Consistently, GATA3 expression was higher in xenografts derived from breast cancer cells with shRNA NC compared with that in lncRNA LOC645166 - silencing group following treatment with adriamycin (Figure 5G). Furthermore, lncRNA LOC645166 overexpression enhanced, while lncRNA LOC645166 knockdown suppressed the luciferase reporter activity of GATA3 (Figure 5H). Together, we hypothesized that lncRNA LOC645166 promotes adriamycin resistance by activating GATA3 transcription.

### Lnc-LOC645166 recruits NF-κB to promote GATA3 transcription

**Figure 6:**
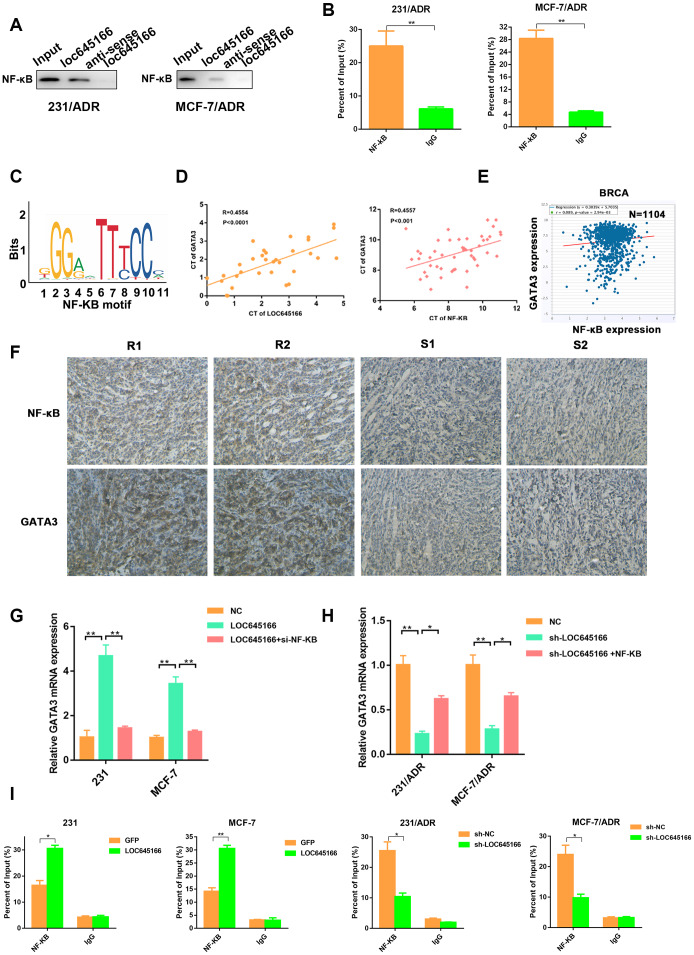
**lnc-LOC645166 recruits NF-κB to promote GATA3 transcription.** (**A**) RNA pulldown assays were performed and the interaction between lnc-LOC645166 and NF-κB was confirmed by Western blot. (**B**) RIP assay of the enrichment of NF-κB with lnc-LOC645166 relative to IgG in the lysates of 231/ADR and MCF-7/ADR cells (n =3). (**C**) NF-κB motif. (**D**) The correlation between lnc-LOC645166 and GATA3 expression was assessed in breast cancer tissues using a Pearson’s correlation analysis. (**E**) The correlation between NF-κB and GATA3 expression was assessed in TCGA Breast Cancer samples using a Pearson’s correlation analysis. (**F**) Representative immunostaining of NF-κB and GATA3 in tumor samples with good or poor responses to adriamycin therapy. R means resistant to ADR therapy, S means sensitive to ADR therapy. (**G**) RT–qPCR analysis of GATA3 in breast cancer cells transfected with pcDNA-LOC645166 or cotransfected with si-NF-κB. (**H**) RT–qPCR analysis of GATA3 in adriamycin resistant cells transfected with sh-LOC645166 or cotransfected with pcDNA-NF-κB. (**I**) Chromatin immunoprecipitation (ChIP) assays were performed to determine the affinity of NF-κB on the promoter region of the GATA3 locus after lnc-LOC645166 overexpression or knockdown. 2% Input cell lysate was used data are shown as means ± SD. *p < 0.05, **p < 0.01, ***p < 0.001.

Recent studies have reported that lncRNAs interact with proteins to regulate target genes [15]. Bioinformatics analysis and literature retrieval suggested NF-κB may function as a mediator among lncRNA LOC645166 and GATA3. Previous studies elucidated that NF-κB was associated with ADR tolerance and breast cancer tumourigenesis [16–18]. Moreover, NF-κB binds to lncRNAs to serve as an oncogene [19, 20]. We performed a western blot assay in the RNA pull-down precipitates with lncRNA LOC645166 biotin-labelled and lncRNA LOC645166 antisense biotin-labelled. We determined that NF-κB could bind with lncRNA LOC645166 (Figure 6A). In addition, RNA Immunoprecipitation (RIP) assay verified the interaction between lncRNA LOC645166 and NF-κB. JASPAR database (http://jaspar.genereg.net/cgibin/jaspar_db.pl) predicted that an NF-κB motif (Figure 6B) could bind to the promoter of *GATA3*. The expression of NF-κB was positively correlated with the expression of *GATA3* in clinical breast cancer tissues and TCGA data (Figure 6D, 6E). In addition, correlation of NF-κB and GATA3 expressions was confirmed in consecutive human breast cancer tissues by IHC analysis (Figure 6F). Moreover, we found that the level of GATA3 was elevated by lncRNA LOC645166, while *NF-κB* knockdown partially attenuated the effects of lncRNA LOC645166 overexpression. Furthermore, *NF-κB* overexpression could rescue the downregulated level of *GATA3* by lncRNA LOC645166 silencing (Figure 6G, 6H). As expected, lncRNA LOC645166 silencing decreased the binding activity of NF-κB on the *GATA3* promoter; whereas, the recruitment of NF-κB to the promoter of *GATA3* increased when lncRNA LOC645166 was overexpressed (Figure 6I).

### Lnc-LOC645166 promoted breast cancer chemoresistance by binding NF-κB to increase the expression of GATA3

**Figure 7:**
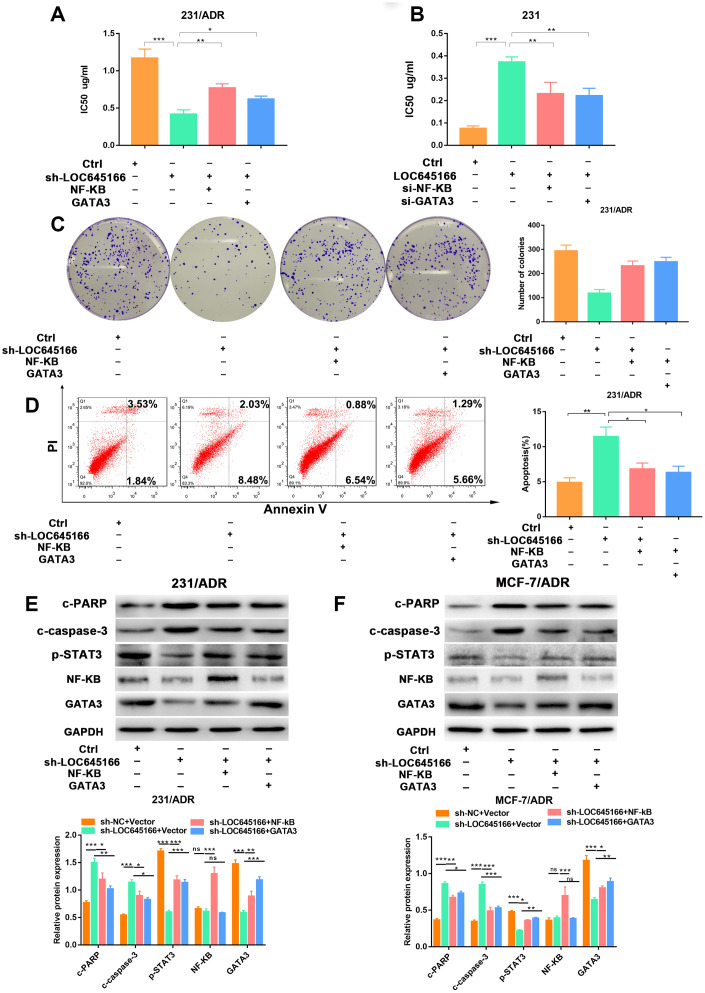
**The biological role of lnc-LOC645166 in breast cancer cells was mediated by NF-κB and GATA3 and E2F3.** (**A**) CCK-8 assays were performed to determine the ADR sensitivity of MCF-7/ADR cells cotransfected with sh-LOC645166 and pcDNA-NF-κB or pcDNA-GATA3. (**B**) CCK-8 assays were performed to determine the ADR sensitivity of 231 cells cotransfected with lnc-LOC645166 and si-NF-κB or si-GATA3. (**C**) Colony formation ability of 231/ADR cells was assessed when cotransfected with sh-LOC645166 and pcDNA-NF-κB or pcDNA-GATA3. (**D**) The apoptosis rate of 231/ADR cell cotransfected with sh-LOC645166 and pcDNA-NF-κB or pcDNA-GATA3 was measured by flow cytometric analysis. (**E**, **F**) Western blot assays were used to measure the expression of NF-κB, GATA3, c-PARP, c-Caspase-3 and p-STAT3 in 231/ADR and MCF-7/ADR cells cotransfected with sh-LOC645166 and pcDNA-NF-κB or pcDNA-GATA3. Data are shown as means ± SD. *p < 0.05, **p < 0.01, ***p < 0.001.

Rescue assays were carried out to demonstrate the role of lncRNA LOC645166/NF-κB/*GATA3* axis in regulating the adriamycin tolerance in breast cancer cells. As shown in Figure 7A, the increased sensitivity of 231/ADR cells by sh- lncRNA LOC645166 was partially attenuated by the overexpression of NF-κB or GATA3. In parent cells, lncRNA LOC645166 overexpression elevated the IC_50_ values, which could be rescued by the downregulation of *NF-κB* or *GATA3*. (Figure 7B).

Furthermore, lncRNA LOC645166 silencing weakened the proliferative ability of 231/ADR cells with ADR treatment, and the introduction of NF-κB or GATA3 could partially reverse this effect (Figure 7C). Additionally, the apoptosis rate increased when lncRNA LOC645166 was knocked down, which could be eliminated in large part by the administration of pcDNA-*NF-κB* or pcDNA-*NF-κB* transfection (Figure 7D, 7E, and Supplementary Figure 4). Besides, the decreased p-STAT3 expression by lncRNA LOC645166 knockdown could also rescued by NF-κB or NF-κB overexpression (Figure 7E).

**Figure 8:**
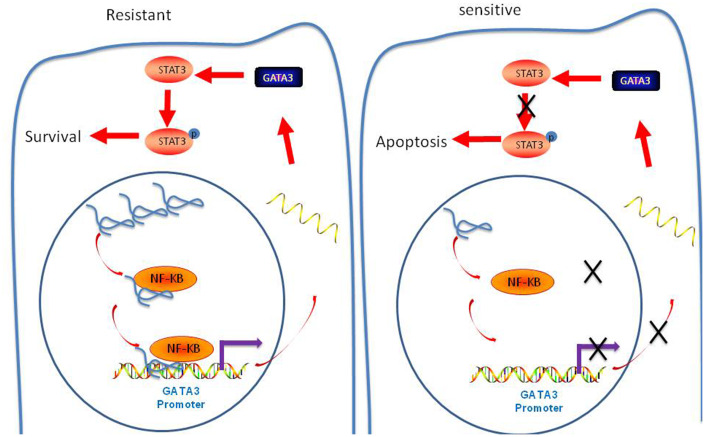
**Schematic diagram of lncRNA-LOC645166-based regulatory mechanism in ADR resistance of breast cancer cells.**

In conclusion, we confirmed that that lncRNA-LOC645166 promotes STAT3 activation by recruiting NF-κB to the promoter region of GATA3, thus eliciting ADR tolerance in Breast cancer cells (Figure 8).

## DISCUSSION

Chemotherapy is the essential strategy for breast cancer treatment [2, 21]. However, the occurrence of nonresponsive chemotherapy has greatly impeded the survival rates. Therefore, a better understanding of the molecular mechanisms involved in drug resistance and identifying predictive biomarkers in breast cancer are highly desired attributes in the field of breast oncology. LncRNAs are becoming valuable molecules as more evidence is demonstrating the number of crucial roles they play in cellular function and oncology. For example, dysregulation of lncRNAs is closely associated with tumourigenesis and chemotherapy resistance in a variety of cancers. LncRNA taurine up-regulated 1 (*TUG1)* has been reported to promote chemoresistance of SCLC by binding the protein enhancer of zeste 2 polycomb repressive complex2 subunit (EZH2) to epigenetically regulate *LIMK2b* expression [22]. Lnc *ARSR* was highly expressed in renal tumour-initiating cells (T-ICs) and contributed to tumourigenesis and drug resistance via binding to yes-associated *protein* 1(*YAP*) and facilitated *YAP* nuclear translocation [15].

In this study, we used microarray analysis to identify an adriamycin resistance-related lncRNA in the paired adriamycin-resistant and sensitive breast cancer cell lines, which was first reported by our previous work. We confirmed lncRNA LOC645166 as being highly expressed in adriamycin-resistant breast cancer cell lines and tissues, suggesting that lncRNA LOC645166 might be involved in the mechanisms underlying adriamycin resistance. By applying gene intervention technology to decrease or increase lncRNA LOC645166 expression, the sensitivity of breast cancer cells to adriamycin was markedly altered. Knockdown of lncRNA LOC645166 also re-sensitised the MCF-7/ADR cells to adriamycin based on the **in vivo** study. Mechanistically, lncRNA LOC645166 recruits NF-κB to the promoter region of *GATA3* and promotes its transcription, eliciting adriamycin resistance in breast cancer cells. Our research demonstrated how lncRNA LOC645166 plays a role in chemoresistance and may be an ideal biomarker in predicting the response to adriamycin in patients with breast cancer.

To clarify the mechanisms underlying the functional role of lncRNA LOC645166 in adriamycin resistance, we screened for potential interaction transcription factors that met the following requirements: Firstly, it is associated with tumourigenesis and chemotherapy resistance in breast cancer; Secondly, it can potentially regulate transcription of *GATA3* and finally, it can be bound to lncRNA. Through bioinformatics analysis and literature retrieval, we found NF-κB might be the potential protein that interacts with lncRNA LOC645166. We performed RNA pull-down assays and verified biotin-labelled lncRNA LOC645166 bound NF-κB, as detected with western blotting. RNA immunoprecipitation (RIP) assays further exhibited the direct interaction between NF-κB and lncRNA LOC645166 in breast cancer cells. These results confirmed that lncRNA LOC645166 binds to NF-κB directly. A previously reported lncRNA, lncRNA *SRLR*, has been identified as being highly expressed in patients with RCC and closely related to intrinsic sorafenib resistance. Mechanistically, lncRNA *SRLR* could specifically recruit NF-κB to promote *IL-6* transcription, thus activating the STAT3 pathway. LncRNA *NKILA* has been reported to directly bind to NF-κB, acting as a modulator to block the activation of the NF-κB pathway and suppress breast cancer metastasis. These studies inspired us to investigate if NF-κB might be the mediator of lncRNA LOC645166 on regulating *GATA3*, and several related experiments further verified this potential mediator.

Giving the evidence that the activation of survival signaling pathways, such as JAK-STAT3 signaling, might contribute to drug tolerance [23]. Therefore, we detected the phosphorylation of STAT3 level when lncRNA LOC645166 was overexpressed or silenced. Our results agree with the of previous findings on hepatocellular carcinoma and breast cancer showing that STAT3 activation contribute to the adriamycin resistance [24, 25]. As shown by western blotting assays, STAT3 was activated in lncRNA LOC645166-overexpressing breast cancer cells and was suppressed in lncRNA LOC645166-silencing breast cancer cells under ADR treatment. Furthermore, lncRNA LOC645166 could specifically recruit NF-κB to promote *GATA3* transcription, thus activating the STAT3.

In conclusion, we have shown that lncRNA LOC645166 and *GATA3* are up-regulated in adriamycin-resistant breast cancer cell lines and tissues. In addition, lncRNA LOC645166 exerts these functions by binding NF-κB to the promoter region of *GATA3*, thereby increasing *GATA3* expression and promoting breast cancer chemoresistance. Our results indicate that lncRNA LOC645166 /NF-κB/ GATA3 might be a candidate prognostic biomarker and a target for reversing adriamycin resistance in breast cancer.

## MATERIALS AND METHODS

### Cell lines and culture

Human Breast cancer cell lines MDA-MB-231(231) and MCF-7 were purchased from Tumor Cell Bank of the Chinese Academy of Medical Science (Shanghai, China). MCF-7 adriamycin resistant Breast cancer cell lines (MCF-7/ADR) and MDA-MB-231 adriamycin resistant Breast cancer cell lines (231/ADR) were established from parental MCF-7 and MDA-MB-231 cells and were preserved in 1mg/L and 0.5mg/L ADR (final concentration), respectively. All cell lines were cultured in Dulbecco's Modified Eagle's medium (GIBCO, USA) supplemented with 10% fetal bovine serum (GIBCO, USA), streptomycin (100 mg/mL), and ampicillin (100 U/mL) at 37°C in humidified air with 5% CO_2_.

### Cell transfection

The full length LOC645166, NF-κB, GATA3 were synthesized by GenePharma (Suzhou, China) and cloned into pcDNA3.1(Invitrogen). ALL siRNA was constructed by RiboBio (Guangzhou, China). Breast cancer cell lines were seeded in six-well plates and transfected with pcDNA (4μg) or siRNA (100nM) when reaching 50% density using Lipofectamine 3000 (Invitrogen), according to the manufacturer’s protocol. All siRNA sequences are listed in Supplementary Table 1.

### Construction of stable LOC645166-Silenced cell lines

The expression of lncRNA LOC645166 was stably decreased by LOC645166-targeting short-hairpin (sh)RNAs, the shRNA sequences were introduced and cloned into pLKO.1-puro vector (Sigma-Aldrich) and were then co-transfected into 293T cells with the VSVG and PSPAX packaging plasmid (Addgene, Cambridge, MA, USA) using Lipofectamine 3000 reagent (Invitrogen). Sequences of shRNA Against Specific Target were listed in Supplementary Table 2. The supernatants was harvested and used to infect 231/ADR and MCF-ADR cells, stable clones were screened with puromycin (2 mg/mL) for 4 weeks.

### RT-qPCR analysis

Total RNA was extracted from tissues or cells using TRIzol reagent (Invitrogen) according to the manufacturer’s guidance. RNA from each sample were reverse transcribed into the cDNA using a primerScript RT reagent kit (Takara, Dalian, China). qPCR analyses were performed by SYBR Prime Script RT-PCR kits on the basis of the manufacturer’s protocols. All mRNA expression was calculated with 2^-ΔΔCT^ method and were normalized to GAPDH expression. All assays were carried out independently three times. All the sequences of primers used for RT-qPCR in this study were listed in Supplementary Table3.

### Cellular fractionation assay of LncRNA

Cytoplasmic and Nuclear RNA Purification Kit (Norgen Biotek Corp, Cat. 21000) was used to isolate all sizes of RNA from the cytoplasmic and nuclear RNA fractions according to the manufacturer’s instructions. The U6 RNA was used as nuclear control and GAPDH mRNA as cytoplasmic control. Cellular fractionation assay was verified in two breast cancer cell lines.

### Colony formation assay

Cells were seed into 6-well plates (500 cells/well) and incubated in Dulbecco's Modified Eagle's medium (GIBCO, USA) with 10% FBS at 37°C. 14 days later, the cells were fixed with 4% paraformaldehyde and stained with Giemsa (Sigma-Aldrich, USA) for 40 min. Colony formation ability was determined by counting the number of visible colonies manually. All samples were assayed in triplicate.

### Flow cytometric analysis

Flow cytometry analysis was carried out as previously reported [26]. To analyze the cell apoptosis, Transfected cells were treated with or without drugs for 24 h first. Cell apoptosis assays was detected by an annexin V/propidium iodide detection kit (KeyGen Biotech Co., Nanjing, China) according to manufacturer’s instruction. All experiments were carried out independently three times.

### Caspase-3 activity analysis

The lysate of treated cells was collected, and the activity of caspase-3 was examined using the colorimetric caspase3 assay kit (Beyotime, Jiangsu, China) according to the manufacturer’s instructions.

### *in vitro* chemosensitivity assay

Chemosensitivity was examined through CCK-8 (Sigma, Natick, MA, USA) assays as previously described [26]. Cells in different groups were incubated in 96-well plates under the treatment of indicated concentration of ADR. After 48 h, the absorption of the cells was measured using a CCK-8 kit (Dojindo, Japan) at 450nm (OD450). All experiments were carried out independently three times.

### Western blot

Equivalent amounts of proteins lysates were separated by 10% SDS-PAGE, transferred to 0.22μm PVDF membranes (Millipore, MA, USA), Membranes were blotted blocked in 5% fat-free milk in Tris-buffered saline and Tween 20 (TBST) for 1 h at room temperature, followed by washing and incubated with anti-GATA3 (1:1000, Abcam), anti-Cleaved-PARP (1:1000, Cell Signaling Technology), anti-Cleaved-Caspase3 (1:1000, Abcam), STAT3(1:1000, Abcam), p-STAT3(1:1000, Abcam), anti-NF-κB (1:1000, Abcam) at 4°C overnight. Subsequently, the membranes were incubated with HRP-conjugated IgG for 2h at room temperature and detected with an enhanced chemiluminescence system. A GAPDH antibody was used as control. The experiment was performed in triplicate.

### Human tissue samples

A total of 48 breast cancer freshly-frozen biopsy samples from patients who were diagnosed and underwent surgery were collected for DNA, RNA, or protein extraction. This study was approved by the Institutional Ethical Review Boards of Dongguan People's Hospital of Southern Medical University. Response to ADR of breast cancer patients were determined by computed tomography (CT) or magnetic resonance imaging, clinical progression, or death, with the use of the Response Evaluation Criteria in Solid Tumors (RECIST).

### Microarray analysis

About1×10^6^ MCF-7/ADR and MCF-7 cells were prepared, and total RNA was extracted with TRIzol reagent and purified with a RNeasy mini kit (Cat. # 74106, QIAGEN, Hilden, Germany). Each sample was hybridized with 1.65 μg Cy3-labeled cRNA using a Gene Expression Hybridization Kit (Cat. # 5188-5242, Agilent Technologies) for 17 h and washed in staining dishes (Cat. # 121, Thermo Scientific, Waltham, MA, USA) with the help of a Gene Expression Wash Buffer Kit (Cat. # 5188-5327, Agilent Technologies). Agilent Microarray Scanner (Cat. #G2565CA, Agilent Technologies) was used to scanned then the slides by the default settings: dye channel, green; scan resolution, 3 μm; PMT, 100%; 20 bit. Feature Extraction software 10.7 (Agilent Technologies) was used for data extracting.

### Luciferase reporter assay

PGL3 luciferase reporter vector (Promega) with GATA3 promoter were constructed. Breast cancer cells transfected with sh-LOC645166 or pcDNA-LOC645166 and their corresponding controls were seeded in 6-well plate for 24 h, and then co-transfected with pGL3-basic (2 μg) and Renilla luciferase (20 ng) for 24 h using Lipofectamine 3000 (Invitrogen). Luciferase activity measure was measured using Dual Luciferase Reporter Assay System (Promega). Relative luciferase activity was normalized to Renilla luciferase activity.

### Chromatin immunoprecipitation assays

We harvested transfected Cells for chromatin immunoprecipitation using the EZ-ChIP Chromatin Immunoprecipitation Kit (Millipore, MA, USA) according to the manufacturer’s instruction. The sequences of each primer were shown in supplementary table 4. Immunoprecipitated DNA was quantified using RT-qPCR.

### RNA-pulldown assays

Biotin-labeled lncRNA-LOC645166 full-length sense and antisense were obtained biotin-labeled with the Biotin RNA Labeling Mix (Roche, Mannheim, Germany) and T7 RNA polymerase (Roche). Biotin-labeled DNA was diluted in 10mM Tris pH 7.5, 0.1 M KCl and10 mM MgCl2 were added into protein extract and incubated for 4 h at 4 °C, followed by addition of beads. The beads containing DNA and proteins were then washed three times, then beads were boiled and precipitated proteins were separated by SDS-PAGE and detected by Western blotting analysis.

### RIP assays

A RIP assays were carried out to study whether LOC645166 could interact with NF-κB using a Thermo Scientific RIP kit (Thermo, Waltham, MA, USA) according to the manufacturer’s instructions. NF-κB antibodies were purchased from Abcam and Rabbit IgG (Abcam) was used as the negative control. Purified RNA was subjected to RT-qPCR analysis. The sequences of each primer were shown in supplementary table 5.

### Xenograft transplantation and immunohistochemical assays

Nude mice (5 weeks old) provided by Guangdong Medical Laboratory Animal Center were divided into 4 groups at random and inoculated subcutaneously on the right flank with 2x10^6^ cells MCF-7/ADR cells stably transfected with sh-lnc-LOC645166 or sh-NC. When the tumor size reached approximately 50 mm^3^, adriamycin was given through intraperitoneal injections at a concentration of 5mg/kg; The injection was performed every three days. The animal research protocols associated with the experimental mice were approved by the Dongguan People's Hospital of Southern Medical University. After five weeks, all mice were executed, tumors were excised, paraffin embedded and formalin fixed. Ki-67 and PCNA immunostaining analysis were conducted, TUNEL staining was conducted to detect the apoptosis rate.

For the tumor metastasis models, 2×10^6^ MCF-7/ADR cells stably transfected with sh-lnc-LOC645166 and sh-NC were injected into the tail vein of nude mice and followed by intraperitoneal injection of adriamycin (5mg/kg) or PBS every other day. After an additional 3 weeks, lungs were dissected and metastasis was determined through HE staining.

### Statistical analysis

Data are expressed as means ± SD of three independent experiments. Statistical analyses were performed using SPSS v.21.0 software (IBM, Armonk, NY, USA). Comparisons between two groups were conducted using two-tail Student’s T-test and one-way ANOVA followed by Tukey comparison test in more than three groups. Differences were considered to be statistically significant when P value is less than 0.05.

### Ethics approval

This study was performed in accordance with the ethical standards and the Declaration of Helsinki and according to national and international guidelines. This study has been approved by the ethics committee of Dongguan People's Hospital of Southern Medical University.

## Supplementary Material

Supplementary Figures

Supplementary Tables
