# Supplementary material for: Long noncoding RNA lnc-LOC645166 promotes adriamycin resistance via NF-κB/GATA3 axis in breast cancer
Source: Aging (Albany NY). 2020 May 27;12(10):8893–912. doi: 10.18632/aging.103012 (PMC7288957; doi:10.18632/aging.103012)
Supplement: Supplementary Figures [file aging-12-103012-s002..pdf]

SUPPLEMENTARY FIGURES

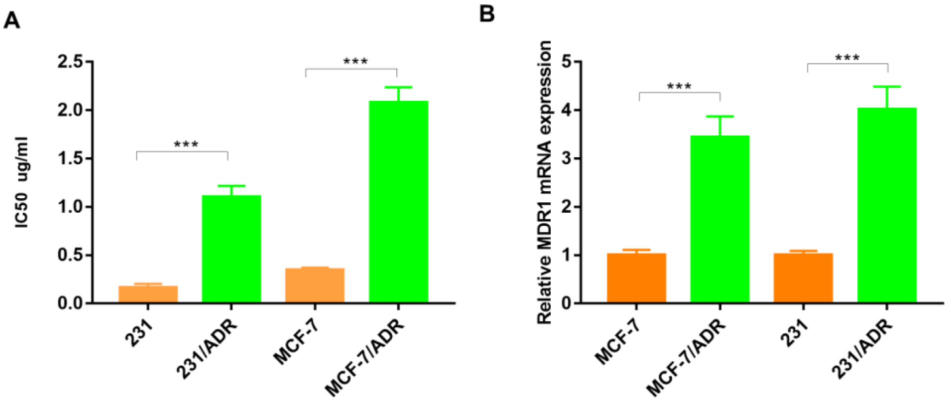

Supplementary Figure 1. (**A**) IC50 values of MDA-MB-231 adriamycin resistant cell (231/ADR), MCF-7 adriamycin resistant cell (MCF-7/ADR) and their parental cells (**B**) MDR1 mRNAs expression in MDA-MB-231 adriamycin resistant cell (231/ADR), MCF-7 adriamycin resistant cell (MCF-7/ADR) and their parental cells. Data are shown as means ± SD. ***p < 0.001.

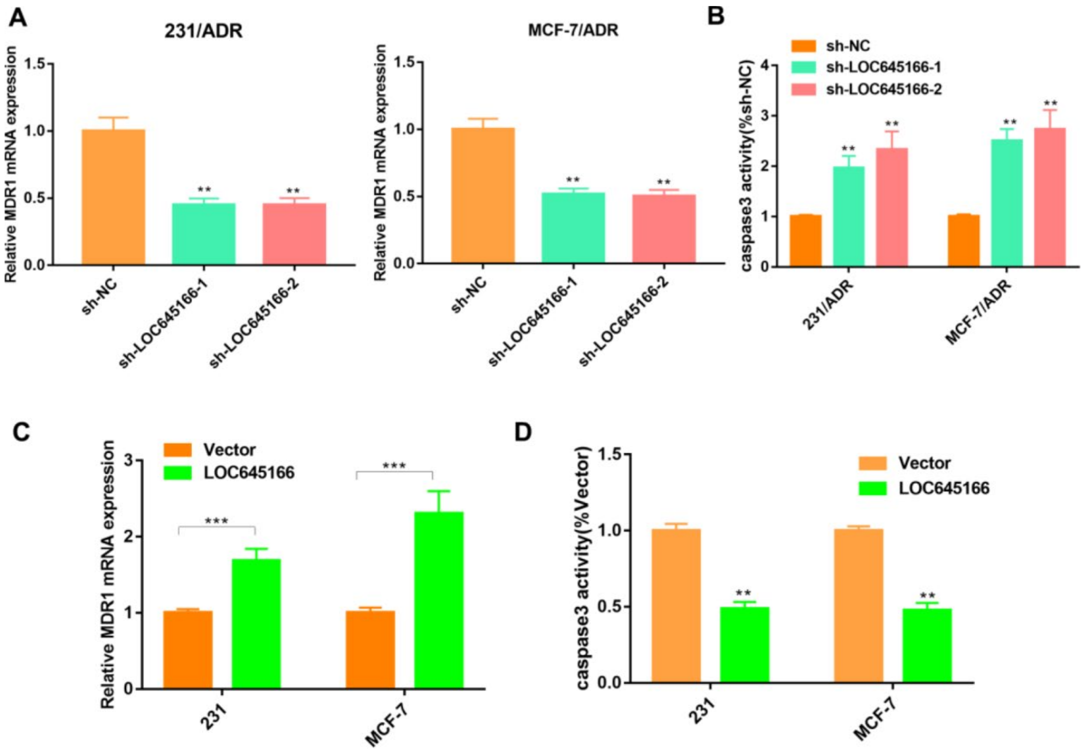

Supplementary Figure 2. (**A**) MDR1 mRNAs expression in the ADR resistance of breast cancer cell lines was detected by RT-qPCR after lnc-LOC645166 was knockdown. (**B**) The activity of caspase-3 was examined in LOC645166 knockdown ADR resistant cell lines using the colorimetric caspase-3 assay kit. (**C**) MDR1 mRNAs expression in the breast cancer cell lines was detected by RT-qPCR when lnc-LOC645166 was overexpressed. (**D**) The activity of caspase-3 was examined in LOC645166 overexpressed cell lines using the colorimetric caspase-3 assay kit. Data are shown as means ± SD. *p < 0.05, **p < 0.01, ***p < 0.001.

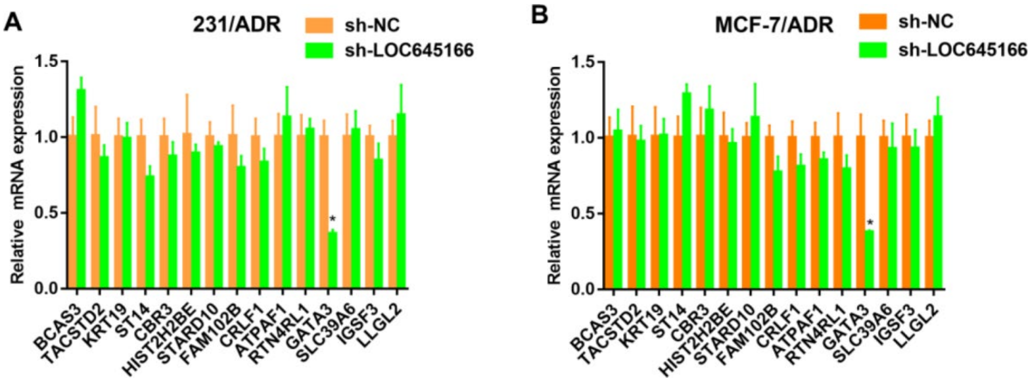

Supplementary Figure 3. (**A**) Different expressed mRNAs in the ADR resistance of breast cancer was detected by RT-qPCR in231/ADR after lnc-LOC645166 was knockdown. (**B**) Different expressed mRNAs in the ADR resistance of breast cancer was detected by RT-qPCR in MCF-7/ADR after lnc-LOC645166 was knockdown. Data are shown as means ± SD. *p < 0.05.

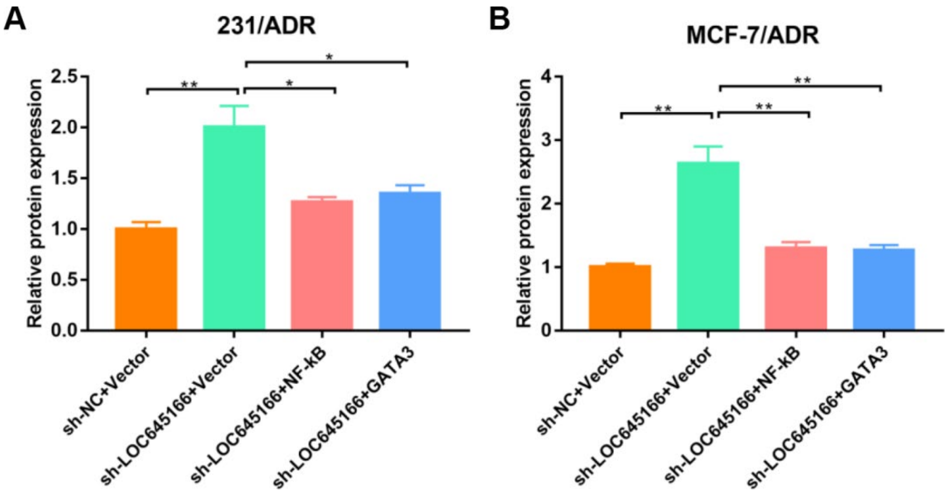

Supplementary Figure 4. The activity of caspase-3 was examined in 231/ADR (**A**) and MCF-7/ADR (**B**) cells cotransfected with sh-LOC645166 and pcDNA-NF-κB or pcDNA-GATA3 using the colorimetric caspase-3 assay kit. Data are shown as means ± SD. *p < 0.05, **p < 0.01.
