# Supplementary material for: Long noncoding RNA lnc-LOC645166 promotes adriamycin resistance via NF-κB/GATA3 axis in breast cancer
Source: Aging (Albany NY). 2020 May 27;12(10):8893–912. doi: 10.18632/aging.103012 (PMC7288957; doi:10.18632/aging.103012)
Supplement: Supplementary Tables [file aging-12-103012-s001..pdf]

SUPPLEMENTARY TABLES

Supplementary Table 1. Sequences of siRNA Against Specific Target.

Si-GATA3	5'-3'	AGCCTAAACGCGATGGATATATG
Si-NF-KB	5'-3'	AACGTAAAAGGACATATGAGACC
sh-NC	5'-3'	TTCTCCGAACGTGTCACGT

Supplementary Table 2. Sequences of shRNA Against Specific Target.

sh-LOC645166-1	5'-3'	GGATCACGAGGTAGGAGTTAA
sh-LOC645166-2	5'-3'	CCAACAGGAGTCTAATCAA
sh-NC	5'-3'	TTCTCCGAACGTGTCACGT

Supplementary Table 3. Sequences for PCR primers used in this study.

ENST00000457075	Forward(5'-3')	GCAGAATGGGGAAATGGAG
	Reverse(5'-3')	AGACAATGGAACAAGCAAACAA
ENST00000452795	Forward(5'-3')	CCTCCGTTCCGTCTATTCTCA
	Reverse(5'-3')	TGGGGCTCTTTCAGGTTCTC
lnc-DIS3-4:1	Forward(5'-3')	TGGGAAGGAAAGCACAATAAC
	Reverse(5'-3')	ACTGGGTAGCAAGGGAACG
ENST00000539086	Forward(5'-3')	CGCCTCTTCTGCATCTTTTA
	Reverse(5'-3')	CGCCGACAGCACTCTTACTA
NR_130928	Forward(5'-3')	GATAAAACGGAAACCCCAGAG
	Reverse(5'-3')	CAGTGAGGGCAAAAGAGGG
lnc-GPR135-3:1	Forward(5'-3')	GCATTATGGTGACTGCCTGTAG
	Reverse(5'-3')	AATATCCTGGCTGCTTGGTTC
Lnc-GGT1-5:2	Forward(5'-3')	TAGGCAAGGACTGGCATCG
	Reverse(5'-3')	AATATCCTGGCTGCTTGGTTC
NR_027356	Forward(5'-3')	CGGTCCCTGGAGGAAACA
	Reverse(5'-3')	CCAACAGTCCCAATAATGCC
NR_037631	Forward(5'-3')	CGGGAAAGGGAAGCAAAG
	Reverse(5'-3')	GAAGACAATGAAAACCCACCAC
ENST00000369605	Forward(5'-3')	CACTGGGAGCCAATGAAACA
	Reverse(5'-3')	CACGAAGTGGAGTTATGGGAAG
BCAS3	Forward(5'-3')	ACTGGGAATGAACCGCCTTTG
	Reverse(5'-3')	CTTCGCCACTGATAGGGATGC
TACSTD2	Forward(5'-3')	ACAACGATGGCCTCTACGAC
	Reverse(5'-3')	GTCCAGGTCTGAGTGGTTGAA
KRT19	Forward(5'-3')	AACGGCGAGCTAGAGGTGA
	Reverse(5'-3')	GGATGGTCGTGTAGTAGTGGC
ST14	Forward(5'-3')	TTCCTGCCAGTCAACAACGTC
	Reverse(5'-3')	GGTACTGCAAATGCCACACC
CBR3	Forward(5'-3')	TGGACATCGACGACTTGCAG
	Reverse(5'-3')	TGTTGACCAGTACGTTGAGCC
STARD10	Forward(5'-3')	TTCCGGTCAGAGTGTGAGG
	Reverse(5'-3')	CGGTACTCAATGTCGTGTAGGAC
FAM102B	Forward(5'-3')	CTTCGTCAATGGGGTCCTCTT
	Reverse(5'-3')	CACAGTTTGCTTGTACCACCT
CRLF1	Forward(5'-3')	CTCTCCCGTGTACTCAACGC
	Reverse(5'-3')	GGGCAGGCCAACATAGAGG
ATPAF1	Forward(5'-3')	CAGTGCATCGCCAACCAAG
	Reverse(5'-3')	TTAAAGGTCTCCACTAACCCGT
RTN4RL1	Forward(5'-3')	GCTGTTGGTAGCTGCGGAG

	Reverse(5'-3')	TCGAGTAGATCCACAGGGTGA
GATA3	Forward(5'-3')	GCCCCTCATTAAGCCCAAG
	Reverse(5'-3')	TTGTGGTGGTCTGACAGTTCG
SLC39A6	Forward(5'-3')	ATGCAAGTCACCACCATAGTCA
	Reverse(5'-3')	ACGTGGAATCAAAATAGGCACT
IGSF3	Forward(5'-3')	AGGGCTCCCACATCACTATCT
	Reverse(5'-3')	GAAGGCAGGTAAATGGACCAC
LLGL2	Forward(5'-3')	CGGGACCTGTTCCAGTTTAAC
	Reverse(5'-3')	CGTCACAGCGTTGTTCTCC
GAPDH	Forward(5'-3')	GGAGCGAGATCCCTCCAAAAT
	Reverse(5'-3')	GGCTGTTGTCATACTTCTCATGG

Supplementary Table 4. Sequences for PCR primers used in ChIP analysis.

GATA3	Forward(5'-3')	CTGGGTCTCAGAGGGTGCT
	Reverse(5'-3')	CGGCTTCTGGTCCTCATTC

Supplementary Table 5. Sequences for PCR primers used in RIP analysis.

LOC645166	Forward(5'-3')	CTGGAAAACAGGAATGTGAGC
	Reverse(5'-3')	CTCTGCGGGTCTGCCATC
